# Integrating a prospective pilot trial and patient-derived xenografts to trace metabolic changes associated with acute myeloid leukemia

**DOI:** 10.1186/s13045-016-0346-2

**Published:** 2016-10-28

**Authors:** Matteo G. Carrabba, Laurette Tavel, Giacomo Oliveira, Alessandra Forcina, Giacomo Quilici, Francesca Nardelli, Cristina Tresoldi, Alessandro Ambrosi, Fabio Ciceri, Massimo Bernardi, Luca Vago, Giovanna Musco

**Affiliations:** 1 Unit of Hematology and Bone Marrow Transplantation, IRCCS San Raffaele Scientific Institute, Via Olgettina 60, 20132 Milan, Italy; 2 Biomolecular Nuclear Magnetic Resonance Unit, IRCCS San Raffaele Scientific Institute, Milan, Italy; 3 Unit of Immunogenetics, Leukemia Genomics and Immunobiology, IRCCS San Raffaele Scientific Institute, Milan, Italy; 4 Molecular Hematology Laboratory, IRCCS San Raffaele Scientific Institute, Milan, Italy; 5 Center for Statistics in Biomedical Sciences, University Vita-Salute San Raffaele, Milan, Italy; 6 University Vita-Salute San Raffaele, Milan, Italy

**Keywords:** Acute myeloid leukemia, Metabolomics, Patient-derived xenografts, Nuclear magnetic resonance

## Abstract

Despite the considerable progress in understanding the molecular bases of acute myeloid leukemia (AML), new tools to link disease biology to the unpredictable patient clinical course are still needed. Herein, high-throughput metabolomics, combined with the other “-omics” disciplines, holds promise in identifying disease-specific and clinically relevant features.

In this study, we took advantage of nuclear magnetic resonance (NMR) to trace AML-associated metabolic trajectory employing two complementary strategies. On the one hand, we performed a prospective observational clinical trial to identify metabolic changes associated with blast clearance during the first two cycles of intensive chemotherapy in nine adult patients. On the other hand, to reduce the intrinsic variability associated with human samples and AML genetic heterogeneity, we analyzed the metabolic changes in the plasma of immunocompromised mice upon engraftment of primary human AML blasts.

Combining the two longitudinal approaches, we narrowed our screen to seven common metabolites, for which we observed a mirror-like trajectory in mice and humans, tracing AML progression and remission, respectively. We interpreted this set of metabolites as a dynamic fingerprint of AML evolution.

Overall, these NMR-based metabolomic data, to be consolidated in larger cohorts and integrated in more comprehensive system biology approaches, hold promise for providing valuable and non-redundant information on the systemic effects of leukemia.

**Electronic supplementary material:**

The online version of this article (doi:10.1186/s13045-016-0346-2) contains supplementary material, which is available to authorized users.

Re-programming energy metabolism is a hallmark of cancer [1]. Acute myeloid leukemia (AML) permits analysis of the impact of a systemic cancer on the metabolism over time. Oncometabolites have been demonstrated in 5–20 % of AML-harboring mutations in Isocitrate Dehydrogenase (IDH) genes [2, 3], and the metabolic profile of AML cell lines can be employed to investigate drug treatments [4, 5]. Moreover, previous studies linked AML with perturbation of metabolic pathways including glucose metabolism [6, 7]. Nuclear magnetic resonance (NMR) is a rapid, highly reproducible cost-effective analytical tool to profile metabolic fluctuations, requiring minimal sample manipulation and well suited for automation and high-throughput purposes, thus ideal for untargeted analysis in clinical applications [8–11].

To study the metabolic changes associated with AML in patients, we enrolled nine newly diagnosed patients in a prospective trial (METAM-02 trial, Additional file 1) and monitored the metabolic trajectory of blast clearance during the first two cycles of intensive chemotherapy (CT) (Fig. 1a). Peripheral blood (PB), bone marrow (BM), and urine samples were collected prior, during, and after induction and consolidation CT, resulting in nine time points (tp_H_) (Fig. 1b). At the final time point (tp9_H_), all patients had achieved complete remission.

**Fig. 1:**
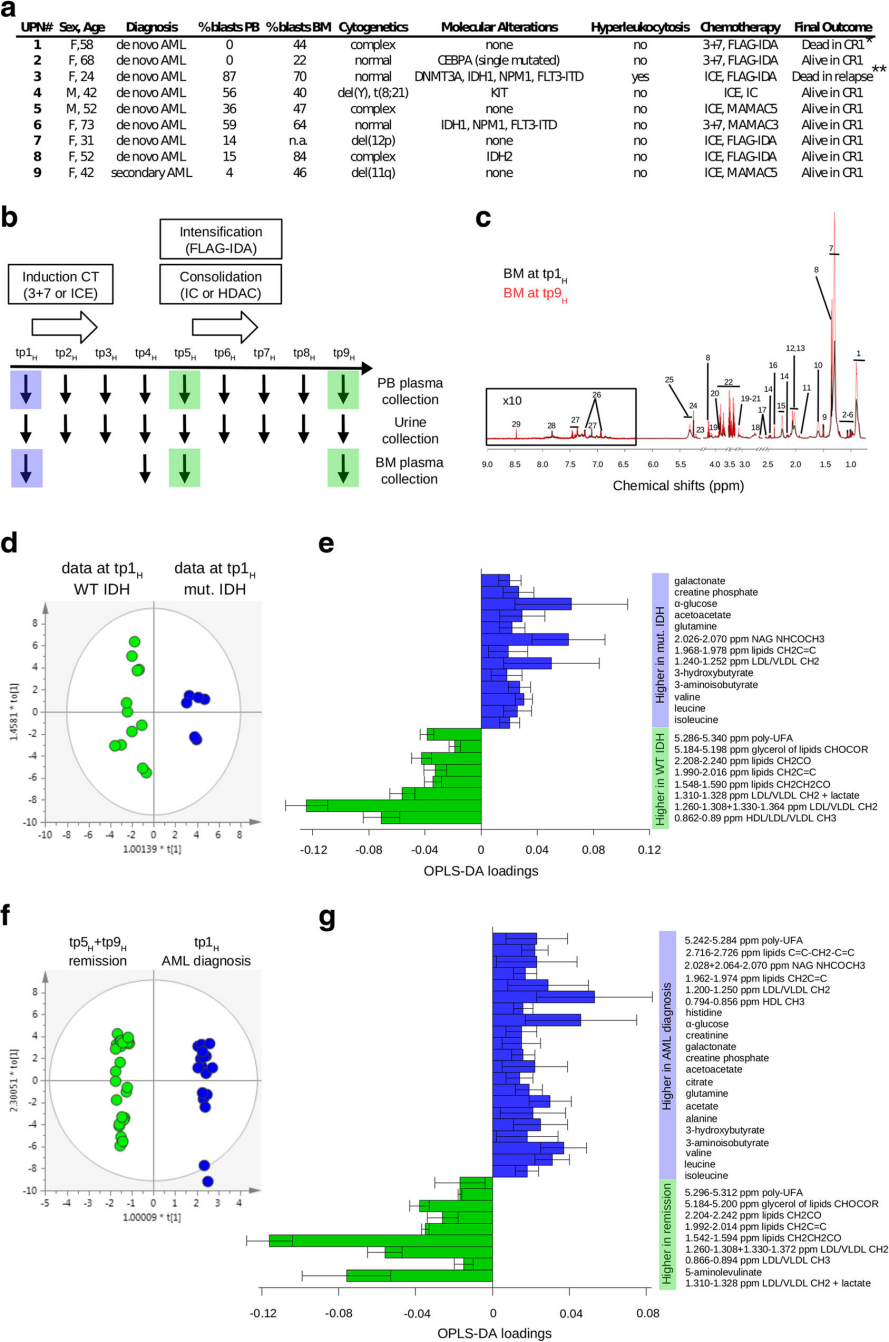
Metabolic changes in AML patients undergoing intensive chemotherapy. **a** Patient characteristics. *CR1* first complete remission. *UPN#1 developed a lethal fungal pneumonia while in remission after the first consolidation CT cycle. **UPN#3 underwent allogeneic hematopoietic stem cell transplantation in CR1 and subsequently relapsed. **b** Outline of the study design. **c** Superposition of representative ^1^H Carr-Purcell-Meiboom-Gill (CPMG) spectra of BM at tp1_H_ (*black line*) and tp9_H_ (*red line*) acquired at 37 °C on a Bruker Avance 600-MHz spectrometer. The ^1^H CPMG NMR experiment is based on a pulse sequence that strongly reduces the NMR signals deriving from large molecules; herewith, molecules with high molecular weight are essentially invisible in the ^1^H spectrum, thus facilitating spectra interpretation and small molecule identification in the presence of large proteins and lipoproteins. Peaks correspond to the different metabolites: *1*—high-, low-, and very low-density lipoproteins (HDL, LDL, VLDL) CH3; *2*—isoleucine; *3*—leucine; *4*—valine; *5*—3-aminoisobutyrate; *6*—3-hydroxybutyrate; *7*—LDL/VLDL CH2; *8*—lactate; *9*—alanine; *10*—lipids CH2CH2CO; *11*—acetate; *12*—lipids CH2C=C; *13*—N-acetyl-glycoproteins (NAG) NHCOCH3; *14*—glutamine; *15*—lipids CH2CO; *16*—acetoacetate; *17*—citrate; *18*—lipids C=CCH2C=C; *19*—creatinine; *20*—creatine; *21*—creatine phosphate; *22*—glucose; *23*—glycerol of lipids CHOCOR; *24*—α glucose; *25*—poly-unsaturated fatty acids (UFA); *26*—tyrosine; *27*—phenylalanine; *28*—histidine; *29*—formate. **d** OPLS-DA score plot for pooled PB and BM samples collected at diagnosis and typing positive (*blue circles*; *n* = 6) or negative (*green circles*; *n* = 12) for missense mutations in the IDH1/2 genes. OPLS-DA with *N* = 18, CV ANOVA *p* = 0.059, *R* ^2^ = 0.93, and *Q* ^2^ = 0.615. The area under the curve (AUC) of the ROC analysis was 0.86 (*p* < 0.001). **e** Metabolites discriminating AML patients with or without IDH gene mutations. Loadings indicate how much the variables, i.e., the metabolites, contribute to the model. Shown are loadings with jack-knifed confidence interval. The metabolites significantly contributing to the model were selected based on variable importance in projection >1 and jack-knifed confidence interval of loadings not crossing the *zero line*. Positive loading values (*blue bars*) indicate the metabolites increased in patients with mutated IDH, while negative values (*green bars*) are associated with increased levels in patients with wild-type IDH. Note that the concentration levels of the classical IDH mutation oncometabolite (R)-2-hydroxyglutarate were below the NMR detection limit and that because of the overlap of the resonances associated to CH2 groups of high-, low- and very low-density lipoproteins (HDL, LDL, VLDL) and the CH2C=C groups of different lipid molecules it was not possible to establish their specific contribution to the model. **f** OPLS-DA score plot for pooled PB and BM samples of patients at diagnosis (tp1_H_, *blue circles*; *n* = 18) vs remission after chemotherapy (pooling samples after induction, tp5_H_, and after first consolidation, tp9_H_, *green circles*; *n* = 29). OPLS-DA model with *N* = 47, CV-ANOVA *p* = 0.006, *R* ^2^ = 0.99, and *Q* ^2^ = 0.671. The AUC of the ROC analysis was 0.73 (*p* < 0.001). **g** Metabolites discriminating AML patients at diagnosis and in remission after chemotherapy. Positive loading values (*blue bars*) indicate the metabolites increased in AML patients at tp1_H_, while negative values (*green bars*) are associated with increased metabolite levels at tp5_H_ + tp9_H_

Seventy-four PB, 33 BM, and 75 urine samples were analyzed by NMR spectroscopy (Fig. 1c and Additional file 1). First, based on results from unsupervised statistical inspection, we decided to pool BM samples with the respective PB and observed that despite the small number of cases, supervised orthogonal partial least squares-discriminant analysis (OPLS-DA) allowed clustering of the samples at diagnosis (tp1_H_) according to the presence or absence of mutations in IDH genes (Fig. 1d, e), supporting the notion that these mutations have an overall impact on the metabolome [2, 3]. Moreover, OPLS-DA discriminated the metabolic differences between patients at disease diagnosis and those in remission (tp5_H_ and tp9_H_), highlighting 30 metabolites significantly contributing to the model (Fig. 1f, g).

To pinpoint the most relevant ones for AML biology, we integrated our study with a mouse-human AML model, allowing us to follow the metabolic changes associated with disease progression. Six littermate NOD/SCID γ-chain-null (NSG) mice were infused with 2 × 10^6^ primary AML blasts (from patient #3). PB samples were collected over time from the same animals before and after AML infusion for a total of nine time points (tp_M_) (Fig. 2a). AML became detectable (blast count > 1 cell/μl) in the PB at tp5_M_ for all infused mice; thereafter, an exponential growth started (Fig. 2b). We thus compared samples before AML infusion (tp1_M_ and tp2_M_) and in the exponential phase of disease (tp5_M_ and tp6_M_). Possibly due to the lower number of variables present in the mouse model, OPLS-DA (Fig. 2c) pinpointed only 22 metabolites accounting for AML progression, mainly involved in pathways related to energy and fatty acid metabolism (Fig. 2d). The increased lactate level in the plasma is a typical hallmark of cancer, characterized by an aerobic glycolytic shift (“Warburg effect”) [1, 12]. Lower plasma levels of cholesterol, lipids, and unsaturated fatty acids were found in AML mice than in healthy controls, in agreement with previous reports [6, 13, 14], highlighting an involvement of fatty acid metabolisms in AML, possibly related to the demand for lipids and cholesterol in tumor proliferation. Also, the increase of glutamine in AML mice is consistent with cancer since glutamine is a major carbon and nitrogen source for tumor cell proliferation [15].

**Fig. 2:**
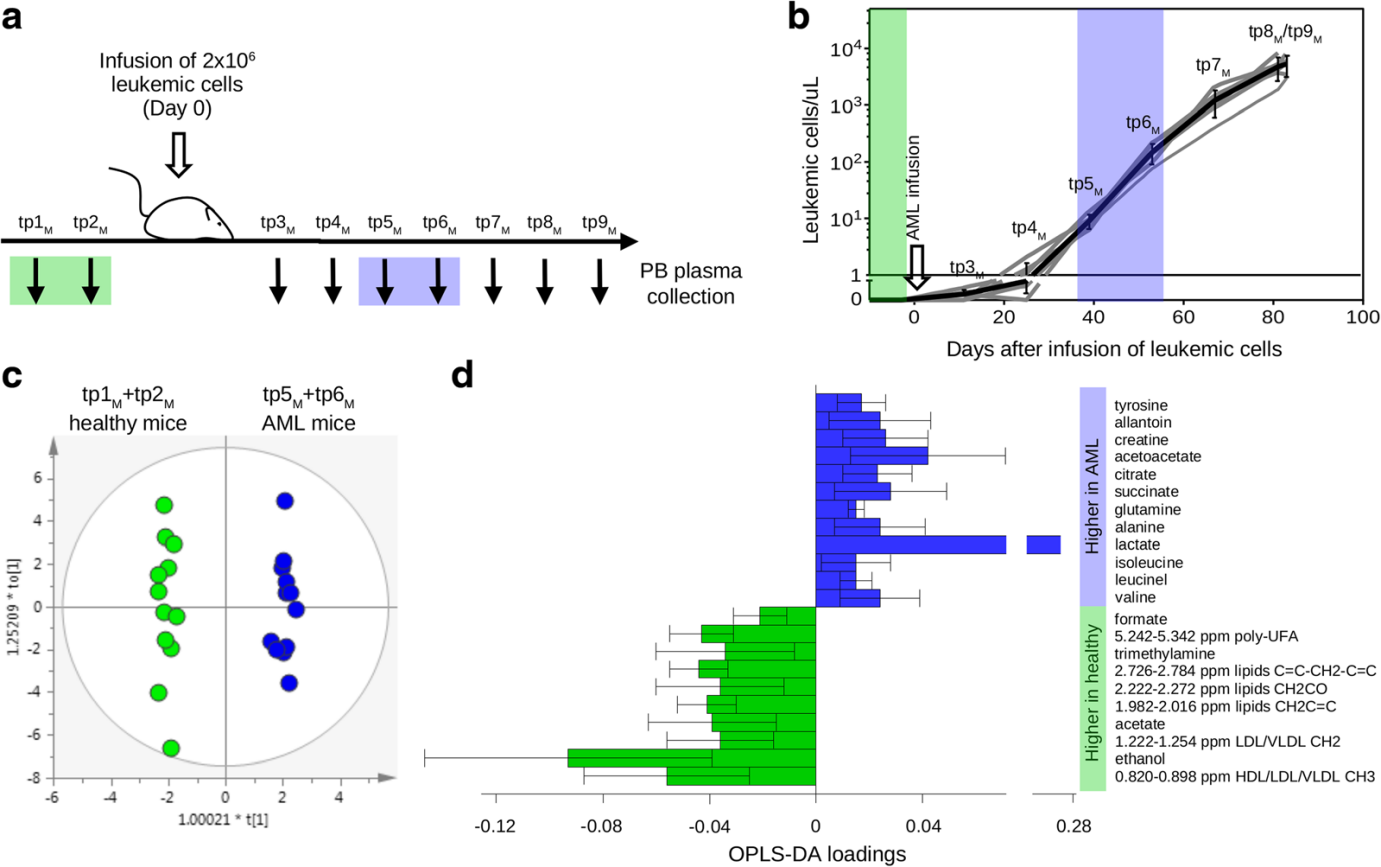
Metabolic profile of the AML mouse-human model. **a** Design of the study. **b** Leukemic cell count (human CD33+, CD45+ values) over time. **c** OPLS-DA score plot for healthy mice (tp1_M_ and tp2_M_; *green circles*; *n* = 12) vs mice at tp5_M_ and tp6_M_ (*blue circles*; *n* = 12) showing an AML metabolic signature at an early stage of AML. OPLS-DA model with *N* = 24, CV-ANOVA *p* = 0.002, *R* ^2^ = 0.99, and *Q* ^2^ = 0.819. The area under the curve (AUC) of the ROC analysis was 0.96 (*p* < 0.001). **d** Metabolites discriminating healthy and human AML-engrafted mice_._ Positive loading values (*blue bars*) indicate the metabolites increased in AML mice, while negative values (*green bars*) are associated with increased levels in healthy mice

Combining the two longitudinal approaches to trace AML in patients and mice, we narrowed our screen to seven metabolites representative of AML tumor burden: leucine, valine, alanine, glutamine, citrate, acetoacetate, and lipids CH2CO. We interpreted this set of metabolites as a dynamic fingerprint of AML evolution that could be followed in a mirror-like trajectory in both studies (Fig. 3).

**Fig. 3:**
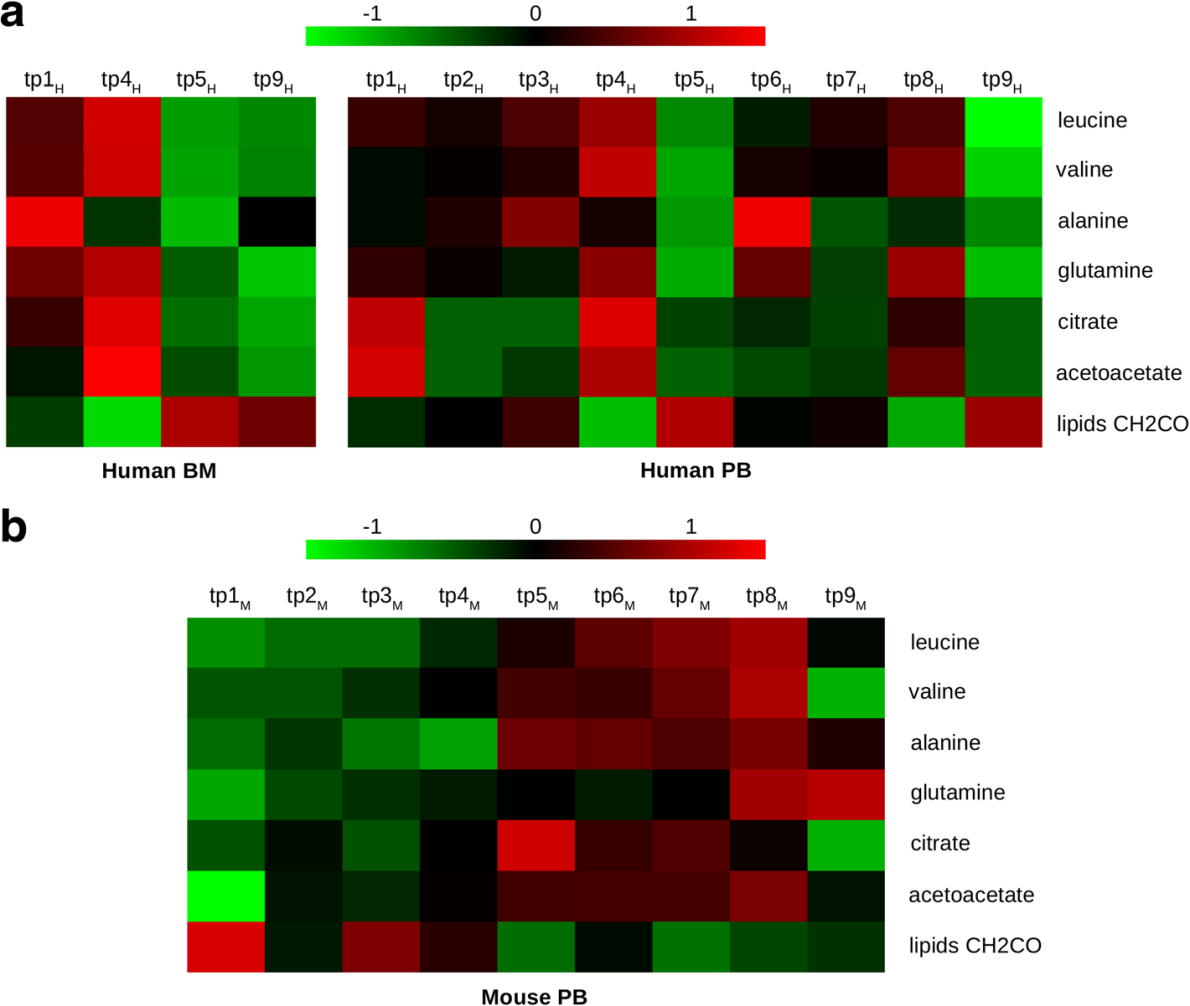
Heat maps of human or mouse plasma tracing the trajectory of the seven metabolites associated with AML evolution in both the patients and mice. **a** Heat maps of human BM and PB tracing the trajectory of the seven metabolites (averaged normalized metabolite area) associated with AML evolution in both the mice and patients. **b** Heat map of mouse PB depicting the trajectory of the seven metabolites (averaged normalized metabolite area of the six mice) associated with AML evolution in both the mice and patients. Note that leucine, valine, alanine, citrate, and acetoacetate increased upon AML progression until tp8_M_ and then decreased at tp9_M_. This reverse effect supported our hypothesis about a metabolic change due to overall systemic failure at a late stage of disease and not to a specific effect of AML

Collectively, our results highlight how NMR-based metabolomics might provide valuable and non-redundant information on the systemic effects of leukemia, to be consolidated in larger cohorts, and integrated in more comprehensive system biology approaches. Although the limited number of cases investigated in this study does not allow a meaningful comparison to previous reports [6, 7], the technical robustness and reproducibility of NMR, together with the minimal sample manipulations and invasiveness of this technique, appear well suited to perform longitudinal studies in clinical settings. Moreover, we first provide experimental evidence on how patient-derived xenografts can be integrated into metabolomic studies, complementing and narrowing the screening of clinically relevant metabolites.

## Additional file

Additional file 1: Supplementary Methods, Results and Figures. (PDF 966 kb)
