# Supplementary material for: Integrating a prospective pilot trial and patient-derived xenografts to trace metabolic changes associated with acute myeloid leukemia
Source: J Hematol Oncol. 2016 Oct 28;9:115. doi: 10.1186/s13045-016-0346-2 (PMC5086061; doi:10.1186/s13045-016-0346-2)

SUPPLEMENTARY METHODS

Clinical Study

The METAM-02 clinical study was a San Raffaele Institutional Review Board-approved prospective, single-arm, observational monocentric trial, aimed at investigating the feasibility of a ^{1}H-NMR based metabolomics approach for longitudinal monitoring of metabolic changes in AML patients receiving first line chemotherapy for AML with curative intent.

Primary endpoints of the trial were 1) to obtain NMR-based metabolic profiles of patients undergoing intensive treatments for AML and 2) to analyze the role of metabolic profiles obtained at specific time-points during induction and consolidation as possible biomarkers.

Inclusion criteria were: 1) age above 18 years; 2) diagnosis of untreated AML including myeloid sarcoma and excluding acute promyelocytic leukemia, or high-risk myelodysplasia (either de novo or secondary to antecedent hematological disease or chemo-radiotherapy for other cancer); 3) adequate sampling for full cytological, cytochemical, cytogenetic and immunobiological disease characterization according to the most recent FAB, EGIL and WHO criteria; 4) ECOG performance status 0-2; 5) signed informed consent. In particular, as per institutional policy, all AML were characterized at diagnosis with standard cytogenetics and molecular analyses to test for the presence of mutations in FLT3 (both ITD and TKD mutations), NPM1, CEBPA, TP53, DNMT3A, IDH1 and IDH2. Cases harboring core binding factor translocations were additionally screened for KIT mutations.

From January 2014 to August 2014 nine consecutive adult patients signed informed consent and were enrolled to the trial. Clinical data and all administered drugs and fluids were registered in dedicated clinical report forms, and the patients were strictly monitored during CT (weight, fluid input and output, temperature) with blood tests taken daily. Patient and disease characteristics, comorbidities, concomitant therapies and clinical outcome were recorded for the purposes of the

study. Main patient and disease characteristics are summarized in Figure 1a.

Human Sample Collection and Processing

Blood and urine samples for ^{1}H-NMR analysis were prepared as described in [1]. Briefly, human blood plasma was collected in EDTA containing tubes (EDTA BD vacutainer®). Clotting time was reduced to 30 min, and the samples were kept on ice. The samples were spun down at 1600g for 15 min and the supernatant collected. Samples were aliquoted into 2 mL cryo-vials with a volume of 0.6 mL and stored at −80°C until until NMR analysis. For NMR analysis plasma samples were prepared adding 50% v/v phosphate buffer (Na_2HPO_4 0.075 M pH 7.4 in 80%H_2O/20%D_2O with DSS 0.08% and NaN_3 0.04%). 600 uL were then transferred into 5 mm SampleJet NMR tubes (96 rack). Human urine samples were spun down and aliquoted into 2 mL cryo-vials with a volume of 1 mL. Samples were stored frozen at −80°C. Before NMR analysis urine samples were thawed and centrifuged at 12000g at 4°C for 5 min. Urine samples were prepared adding 10% v/v phosphate buffer (KH_2PO4 1.5 M pH 7.4 in 80%H_2O/20% D_2O with DSS 0.1% and NaN_3 2 mM). 600 uL were then transferred into 5 mm SampleJet NMR tubes (96 rack).

Mouse Experiments and NMR Sample preparation

NOD/SCID IL2Rγ^{null} mice were purchased from Charles-River Italia and maintained under specific pathogen-free conditions in the research animal facility of San Raffaele Scientific Institute, Milan. All experimental procedures on animals were performed after specific approval by the San Raffaele Institutional Animal Care and Use Committee (IACUC). Six 4-weeks old male NSG mice born from the same female were chosen and maintained in the same cage for two additional weeks before treatment in order to reduce metabolic stress. In the absence of irradiation, mice received the intravenous infusion of $2x10^6$ AML cells derived from primary leukemic cells of patient #3. Mice were sampled two times before treatment (10 and 3 days before infusion of leukemic cells, $tp1_M$ and

$tp2_M$), and every two weeks after the injection of human leukemic cells (day 11, 25, 39, 53, 67 and 81 after the infusion, corresponding to $tp3_M$, $tp4_M$, $tp5_M$, $tp6_M$, $tp7_M$ and $tp8_M$, respectively) and immediately before sacrifice (day 83, $tp9_M$). For one mouse the disease progression was faster as compared to the others, it was therefore sacrificed at $tp8_M$. Hence, the total number of samples was 53. Blood samples were collected in EDTA BD vacutainer® by retroorbital bleeding with a single-use EDTA-rinsed catheter. Tubes were inverted 8-10 times after blood collection. Quantification of blasts circulating in the peripheral blood of experimental mice was determined by FACS after staining of 50uL of blood with a panel of antibodies specific for murine CD45, human CD45 and human CD33 (Biolegend). Blood was diluted in a 3:1 ratio with in house produced Fc-block (2.4G2 hybridoma, ATCC), incubated with antibodies for 15 minutes at room temperature and lysed with ACK (Ammonium-Chloride-Potassium) lysis buffer. After centrifugation to remove cell debris, absolute counts of leukemic cells (human CD45+ CD33+) was obtained adding Flow-Count beads (Beckman Coulter). Samples were acquired immediately on a Navios cytometer (Beckman Coulter) and analyzed with Kaluza software (Beckman Coulter). For plasma preparation, the remaining blood was rapidly processed and centrifuged at 4°C at 1300g for 10 minutes and the supernatant was collected. Residual red blood cells were separated from plasma after having been centrifuged at 16000g at 4°C for 1 min. Plasma supernatants (Volume ≥35 uL) were transferred into 1.5 mL eppendorf tube and stored at -80°C until NMR analysis. NMR plasma samples were prepared by mixing 35 uL of plasma with 90 uL of H_2O and 125 uL of phosphate buffer (Na_2HPO_4 0.075 M pH 7.4 in 80%H_2O/20%D_2O with DSS 0.08% and NaN_3 0.04%). 250 uL were then transferred into 3 mm SampleJet NMR tubes (96 rack).

^{1}H NMR spectroscopy

A total of 235 samples were analyzed by NMR: 74 PB, 33 BM and 75 urine samples for patients and 53 PB samples for mice. All NMR spectra were acquired on a Bruker Avance (Bruker GmbH,

Rheinstetten, Germany) 600 MHz spectrometer equipped with triple-resonance TCI cryoprobe and refrigerated autosampler (SampleJet). Plasma and urine experiments were carried out at 310 K and 300 K respectively. Samples were randomized and acquisitions ran under automation by IconNMR. For each sample one-dimensional (1D) nuclear Overhauser enhancement spectroscopy (NOESY)-presat (noesygppr1d), and Carr-Purcell-Meiboom-Gill (CPMG) were acquired with 32 (human samples) and 128 (mouse samples) scans, 65K complex data points, spectral width of 20 ppm, relaxation delay of 4 s and mixing time of 10 ms for noesy esperiments. All NMR acquisition parameters are detailed in Dona et al.[1]. 1D ^{1}H NMR spectra were processed with Topspin 3.2 (Bruker GmbH, Rheinstetten, Germany) with 0.3 Hz line broadening, and were automatically phased and baseline-corrected. Spectra were referenced to formate at δ8.45 ppm and 1-methylnicotinamide (pH-insensitive metabolite) at δ9.275 ppm for plasma and urine samples respectively[2]. 1D ^{1}H NMR spectra assignment was performed with Chenomx 8.1 (Chenomx Inc., Edmonton, Canada).

For identification purposes we also acquired 2D J-resolved ^{1}H NMR experiments for human samples and natural abundance 2D ^{1}H-^{13}C Heteronuclear Single-Quantum Coherence (HSQC) and ^{1}H-^{1}H TOtal Correlation SpectroscopY (TOCSY) experiments on selected samples. 2D J-resolved ^{1}H NMR experiments were performed with spectral widths of 16.6 ppm and 78 Hz for F2 and F1, respectively, a relaxation delay of 2 s, and 8 scans. We acquired 2D ^{1}H-^{1}H TOCSY experiments with spectral widths of 12 ppm in both dimensions, 40 scans, and a relaxation delay of 2 s. The ^{1}H-^{13}C HSQC data were obtained with spectral widths of 12 and 170 ppm in the direct and indirect dimensions, respectively, a relaxation delay of 1.5 s, and 94 scans.

Statistical Analysis

Post-processing of all the spectra was performed with Mnova 10.0 (Mestrelab Research). DSS, EDTA and water were excluded from 1D ^{1}H NMR spectra of plasma samples. After alignment,

spectra were divided into regular buckets (0.002 ppm). NMR data were then normalized to total area, Pareto scaled and subjected to statistical analysis using Simca 13.0 software (Umetrics, Umea, Sweden). We performed unsupervised principal components analysis (PCA) and supervised orthogonal partial least squares-discriminant analysis (OPLS-DA)[3]. Orthogonal Projection to Latent Structures (OPLS)[4] is a supervised multivariate regression method that finds relations between two matrices (data X and response Y). It allows to understand which variables are more correlated to a response, and to make predictions for new samples. OPLS-DA[3] is a variant of OPLS, used when Y is discrete or categorical, as when discriminating between different classes or groups. Model adequacy was assessed using the cross-validated scores (the goodness of fit R^2 and the goodness of predictivity Q^2), permutations test and cross-validated ANOVA (CV-ANOVA). The variables were ranked according to their contribution to the model using the VIP (variable influence on projection) parameter. VIP>1 was considered relevant. The loading plot with jack-knifed confidence interval displayed the uncertainty of each variable, in this study, those variables with jack-knifed confidence interval across zero were excluded. In addition, Receiver Operating Characteristic (ROC) curve analysis was used for model performance evaluation. We calculated the Area Under the Curve (AUC) using MetaboAnalyst 3.0[5]. Heat maps providing intuitive visualization of the metabolic trajectory of targeted metabolites were generated by MetaboAnalyst (version 3.0).

1D ^{1}H NMR spectra of urine samples were divided into regular buckets (0.02 ppm) after exclusion of DSS, urea and water signals. Then, NMR data were normalized to total area, Pareto scaled and subjected to statistical analysis using Simca 13.0 software (Umetrics, Umea, Sweden).

SUPPLEMENTARY RESULTS

Analysis of patient samples from the METAM-02 trial

A total of 182 human biofluid samples (74 PB, 33 BM and 75 urine) were analyzed by NMR spectroscopy. The spectra of the samples collected during and just after CT ($tp2_H$-$tp4_H$; $tp6_H$-$tp8_H$) were highly influenced by the drugs (e.g pH, spectra alignment, resonances shifts) hampering reliable spectra binning, and thus compromising untargeted profiling. Therefore we focused our analyses on $tp1_{H,}$ $tp5_H$ and $tp9_{H.}$ We first performed an unsupervised Principal Component Analysis (PCA) on samples to have a global system overview. Consistent with the big inter-samples variability (age, gender, AML heterogeneity), PCA was not able to cluster the samples in separate groups. Interestingly, we did not observe appreciable metabolic differences between PB and BM biofluids, as illustrated by the PCA score plot of the nine patients at diagnosis (Figure S1), where PB and BM deriving from the same patient fall in close proximity in the reduced dimensionality space. Further supervised analysis was therefore performed coupling PB to its corresponding BM. Despite the small number of cases, supervised orthogonal partial least squares-discriminant analysis (OPLS-DA) performed at $tp1_H$ was able to cluster patients according to their European Leukemia Net (ELN) risk of relapse (Figure S2).

Conversely, data derived from the matching urine samples could not be fit in any model (data not shown), suggesting that this biofluid is too influenced by inter-individual variability and treatment to provide meaningful results in a limited patient cohort.

SUPPLEMENTARY REFERENCES

1. Dona AC, Jimenez B, Schafer H, Humpfer E, Spraul M, Lewis MR*, et al*. Precision high-throughput proton NMR spectroscopy of human urine, serum, and plasma for large-scale metabolic phenotyping. *Anal Chem* 2014; 86: 9887-94.

2. Bernini P, Bertini I, Luchinat C, Nincheri P, Staderini S, Turano P. Standard operating procedures for pre-analytical handling of blood and urine for metabolomic studies and biobanks. *J Biomol NMR* 2011; 49: 231-43.

3. Bylesjo M, Rantalainen M, Cloarec O, Nicholson JK, Holmes E, Trygg J. OPLS discriminant analysis: combining the strengths of PLS-DA and SIMCA classifcation. *J Chemometrics* 2006; 20: 341-51.

4. Trygg J, Wold S. Orthogonal projections to latent structures (O-PLS). *J Chemometrics* 2002; 16: 119-28.

5. Xia J, Sinelnikov IV, Han B, Wishart DS. MetaboAnalyst 3.0--making metabolomics more meaningful. *Nucleic Acids Res* 2015; 43: W251-7.

SUPPLEMENTARY FIGURE LEGENDS

Supplementary Figure S1. (**a**) Superposition of representative ^{1}H CPMG spectra of paired PB (black line) and BM (red line) for patient #2 at $tp1_H$. (**b**) PCA score plot of PB (black circles) and BM (red circles) for the nine patients at $tp1_H$ showing the close position of PB and PB of the same individual in the reduced dimensionality space. The symbol # indicates the patient number.

Supplementary Figure S2. (a) OPLS-DA score plot for PB and BM samples collected at diagnosis with low (green circles; n=8) *vs* high (blue circles; n=10) European Leukemia Net risk of relapse. OPLS-DA with N=18, CV-ANOVA p=0.033, R^2=0.999 and Q^2=0.898. The area under the curve (AUC) of the ROC analysis was 0.905 (p<0.001). (**b**) Metabolites discriminating AML cases with low and high ELN risk of relapse. Positive loadings values (blue bars) indicate metabolites increased in high risk patients at $tp1_H$, while negative values (green bars) are associated to increased levels in low risk patients.

SUPPLEMENTARY FIGURES

S1

a

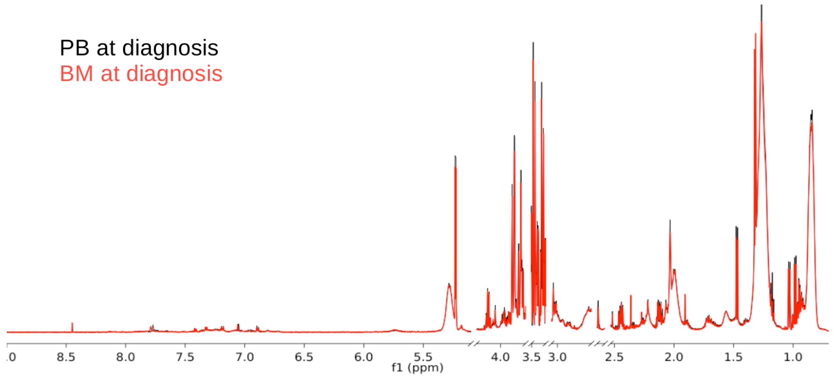

b

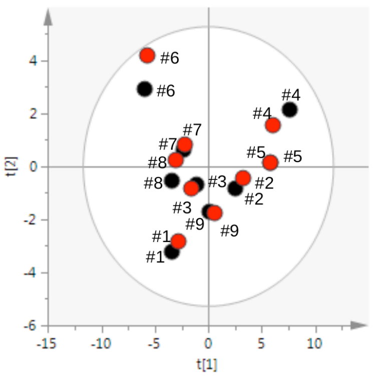

S2

a

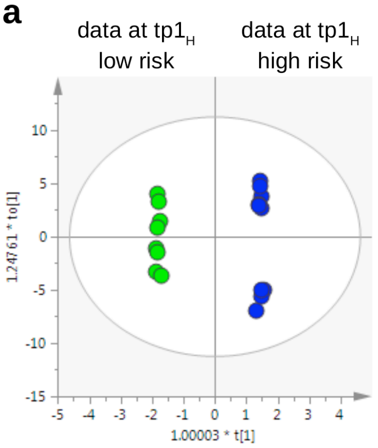

b

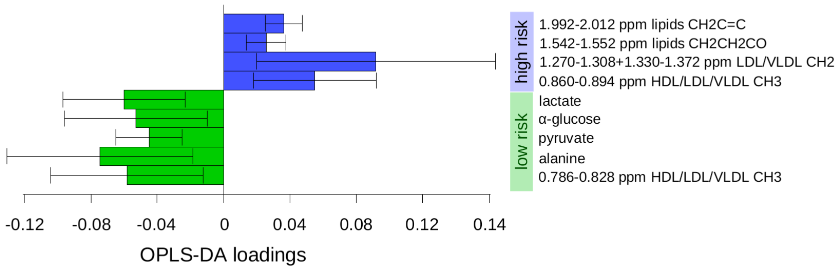

Supplement: Additional file 1: — Supplementary Methods, Results and Figures. (PDF 966 kb) [file 13045_2016_346_MOESM1_ESM.pdf]
